# Spatiotemporal changes of cultivated land utilization in black soil region, China based on geo-information Tupu

**DOI:** 10.1038/s41598-025-95047-1

**Published:** 2025-03-25

**Authors:** Dan Li, Guizhen Chen, Zhilong Xi, Pengkai He, Xiuwen Xin, Jiayuan Chen, Hongyu Yu, Guangqing Kang

**Affiliations:** 1 College of Surveying and Mapping Engineering, Heilongjiang Institute of Technology, Harbin, 150050 China; 2 Institute of Economic Management Science, Ministry of Natural Resources (Heilongjiang Provincial Research Institute of Surveying and Mapping), Harbin, 150081 China; 3 College of Mechanical and Electrical Engineering, Northeast Forestry University, Harbin, 150040 China; 4 School of Environment and Spatial Informatics, China University of Mining and Technology, Xuzhou, 221116 China; 5 School of Earth Sciences, Northeast Petroleum University, Daqing, 163318 China; 6 The Second Institute of Geographic Information Mapping, Ministry of Natural Resources (The Fifth Institute of Surveying and Mapping Geographic Information Engineering of Heilongjiang Province), Harbin, 150081 China

**Keywords:** Geo-information Tupu, Cultivated land utilization, Spatiotemporal variation, Black soil region, Northeast China, Agroecology, Environmental monitoring

## Abstract

In recent years, quantity and quality of cultivated land in black soil region in Northeast China have changed with the continuous strengthening of the breadth and depth of land development, directly affecting food security in China. In this study, land-use data based on geo-information Tupu in 1990, 2000, 2010 and 2020 were selected and the spatiotemporal evolution of cultivated land in Northeast China was analyzed. Meanwhile, driving factors affecting the changes of cultivated land over the past 30 years was also explored. The results indicate that the net increase of cultivated land in the study area is 127.72 km^2^ (accounting for 0.038% of the study area) from 1990 to 2020, and a trend of “increase-decrease-increase” over the past 30 years can be obtained. The change of cultivated land mainly due to the mutual conversion between cultivated land and forest (net increase: 6024.76 km^2^), grassland (net increase: 734.08 km^2^) and construction land (net decrease: 7393. 42 km^2^). The conversions of cultivated land and forest, grassland and construction land are mainly located in the northeastern, mid-western and southern, and eastern of the study area, and the center of cultivated land within the study area shifted towards the southeast from 1990 to 2010, while the center showed a convoluted trend (moving towards the northwest) during the 2010–2020 period, but the migration speed gradually slowed down. The spatiotemporal pattern changes of cultivated land in the study area from 1990 to 2020 are the comprehensive effects of natural environmental and socio-economic factors, among which slope, elevation, and annual precipitation are the main contributing factors. The conclusion of this study will provide scientific reference for the study of cultivated land utilization and protection in the black soil region in Northeast China.

## Introduction

Black soil refers to the land that is rich in organic matter compounds and humus top-soil, which has good properties and high fertility. Owing to the abundant organic matter content and outstanding structure, making the black soil one of the most suitable land types for cultivation^1–3^. Specially, a large amount of nutrients such as N, P and K are contained in the black soil, which are essential for plant growth, and then the top-notch level of fertility will be obtained after years of accumulation. Besides, the excellent soil permeability and strong water retention make it one of the most suitable land-use types that is conducive to plant growth^4,5^. At present, there are three major black soil regions in the world, namely the Great Plains of Ukraine (GPU), the Mississippi River basin in central and northern America (MRBA), and the Northeast Plain of China (NPC)^6,7^. The GPU, which is known as the “granary” of Europe, is the largest black soil region in the world, and the MRBA regarded as the “bread basket” is the second largest distribution area for black soil in the world with a vast territory and a sparse population (5 people per square kilometer)^8,9^. There are numerous farms distributed throughout the MRBA region, leading to this region a typical area for “Commodity Grain Agriculture”^10^. The third largest black soil region in the world, mainly distributed in the Northeast Plain of China^11,12^. The Northeast region of China was once a vast marsh wetland, and the thick layer of black soil gradually generated due to the erosion caused by the peeling of dead branches and fallen leaves, making this region the largest production base for commodity grain in China and playing a crucial role in ensuring national food security in China^13–17^. However, the ecological environment and organic matter content of black soil in Northeast China have suffered a constantly deteriorating with the continuous strengthening of the breadth and depth of land-use development^18–21^. Among all types of damage, the most representative is soil erosion on sloping farmland, and the serious soil erosion may be mainly caused by severe wind erosion, water erosion and freeze-thaw cycle erosion^22,23^. Previous studies have demonstrated the relationship between erosion and soil fertility, and reasons for this adverse impact are also analyzed. The results indicate that the erosion process can effect the physical and chemical properties including soil thickness, organic carbon content, texture, structure and moisture content, and ultimately lead to a decrease in soil fertility and quality^24,25^. In addition, human activities and land-use change caused by anthropogenic factor is another reason affecting soil quality, among which the excessive use of pesticides and fertilizers will accelerate the reduction of black soil layer thickness, leading to the deterioration of soil quality and decrease of soil fertility. Meanwhile, the urbanization and industrialization process has also led to changes in land-use types, directly resulting in compaction and decreasing permeability of soil and the decline in yield of black soil cultivated land^26–28^.

The protection of black soil in Northeast China has received high attention, and the “Outline of Protection Plan for Black Soil in Northeast China (2017–2030)” was formulated in 2017, aiming to effectively protect the sustainable development of agricultural production in black soil region in China^29^. In addition, the “Three Year Action Plan for Protecting Black Soil Farmland in Heilongjiang Province (2018–2020)” had also been formulated and promulgated by the Heilongjiang Provincial People’s Government in 2018, whose main purpose was to effectively improve the quality of cultivated land by adjusting the structure of cultivated land, upgrading farmland facilities and mobilizing farmers’ enthusiasm^30,31^. Meanwhile, the Jilin Provincial People’s Government also issued the “Implementation Plan for Black Soil Protection in Jilin Province” in 2021, emphasizing that it was necessary to consider types of cultivated land, climate conditions, cultivation modes and other factors comprehensively and protect black soil in Jilin Province according to local conditions^32^. It is worth noting that the “Black Soil Protection Law of the People’s Republic of China” was formulated in 2022 and began to implement on August 1, 2022, giving legal significance to the protection of black soil in China for the first time^33^. The fundamental purpose of this law includes protecting black soil resources, restoring and improving the basic fertility of black soil steadily, promoting sustainable utilization of resources, maintaining ecological balance and ensure national food security^34–36^.

Cultivated land, which is regarded as an important agricultural resource, has always been described as a “lifeblood” for its important role in food production^37^. Exploring the evolution of spatiotemporal pattern of cultivated land in black soil region can greatly promote the understanding of the current utilization status of cultivated land and identify the changing characteristics of cultivated land, which will have important practical significance for balancing the utilization and protection of cultivated land. The study of spatiotemporal changes of cultivated land in black soil areas has always been a hot topic, and some scholars in China have conducted extensive researches on the practical problems faced by cultivated land utilization in black soil regions. Li et al. (2021) used GIS technology and the “Geographic Detector” method to analyze the spatiotemporal changes of cultivated land in Heilongjiang Province from 1980 to 2015, and the driving factors leading to spatiotemporal heterogeneity were compared and analyzed quantitatively^38^. Meanwhile, the quality evaluation index system for cultivated land in the black soil region of Northeast China was constructed by means of GIS technology, and the quality of cultivated land in black soil region in Northeast China has also been evaluated, aiming to grasp the spatiotemporal changes of cultivated land quality and explore the reasons for these changes^39^. Zhang et al. (2023) conducted the research on the changes in the quantity and quality of cultivated land in black soil region in Northeast China, summarizing the current situation of utilization and protection for cultivated land in black soil region, and the methods for sustainable development and protecting cultivated land were proposed accordingly^40^. Although the above researches have laid a good foundation for the study of cultivated land utilization in black soil regions, their study areas often didn’t cover all of the black soil regions in China, and there was also a lack of long-term time span, resulting in the inability to reveal the spatiotemporal evolution of cultivated land in black regions in China and explore the driving factors responsible for the spatiotemporal heterogeneity.

The geo-information Tupu is a new method that combines modern technology with traditional research results to explore and analyze the spatiotemporal changes of geographic information data, and the fundamental purpose of geo-information map is to express the temporal evolution and spatial differentiation of natural processes and socio-economic development on a regional scale^41–45^. The core idea of this method is to explore the spatiotemporal knowledge and laws of geoscience, mainly including the inversion of the past, the evaluation of current situation and the prediction of the future, aiming to provide application services for social development and economic construction^46^. Due to the integration of the simplicity of landscape comprehensive maps and the abstraction of mathematical models, this technology is a highly operational and practical decision support system, and has been widely used since its inception. Pradhan et al. (2010) constructed an advanced fuzzy logic model using geographic information data to map the geographical locations of landslide areas, and the accuracy of constructed model was verified by comparing with the logistic regression model^47^. There are also many studies on monitoring land use/land cover (LULC) change using geographic information Tupu. Multi-temporal satellite data was regarded as the foundation and the geo-information Tupu was used to monitor the LULC changes and urban sprawl in Peshawar City in Khyber Pakhtunkhwa in the research conducted by Raziq et al. (2016)^48^. Appiah et al. (2015) attempted to explore the synergy of geo-information Tupu and satellite remote sensing data to analyze the changes of LULC in a Peri-Urban District of Ghana from 1986 to 2016, and the driving factors leading to these changes were compared and analyzed simultaneously^49^.

To our knowledge, there have been few prior studies on the spatiotemporal changes of cultivated land in black soil regions distributed in Northeast China over the past 30 years (1990–2020) and analyzing the driving factors leading to the spatiotemporal heterogeneity. The specific objectives of this study are as follows:

1. Explore the characteristics of quantity change and spatial distribution for cultivated land in black soil region in Northeast China based on land-use data and corresponding theory of geo-information Tupu.
2. Compare and analyze the driving factors that lead to spatiotemporal heterogeneity of cultivated land in black soil regions in Northeast China over the past 30 years (1990–2020) quantitatively based on the PLUS model.
3. Provide theoretical basis for the effective allocation of utilization space for cultivated land, as well as reference for the sustainable use of limited black soil resources and ensuring food productivity in black soil regions in Northeast China.

## Materials and methods

### Study area

**Fig. 1:**
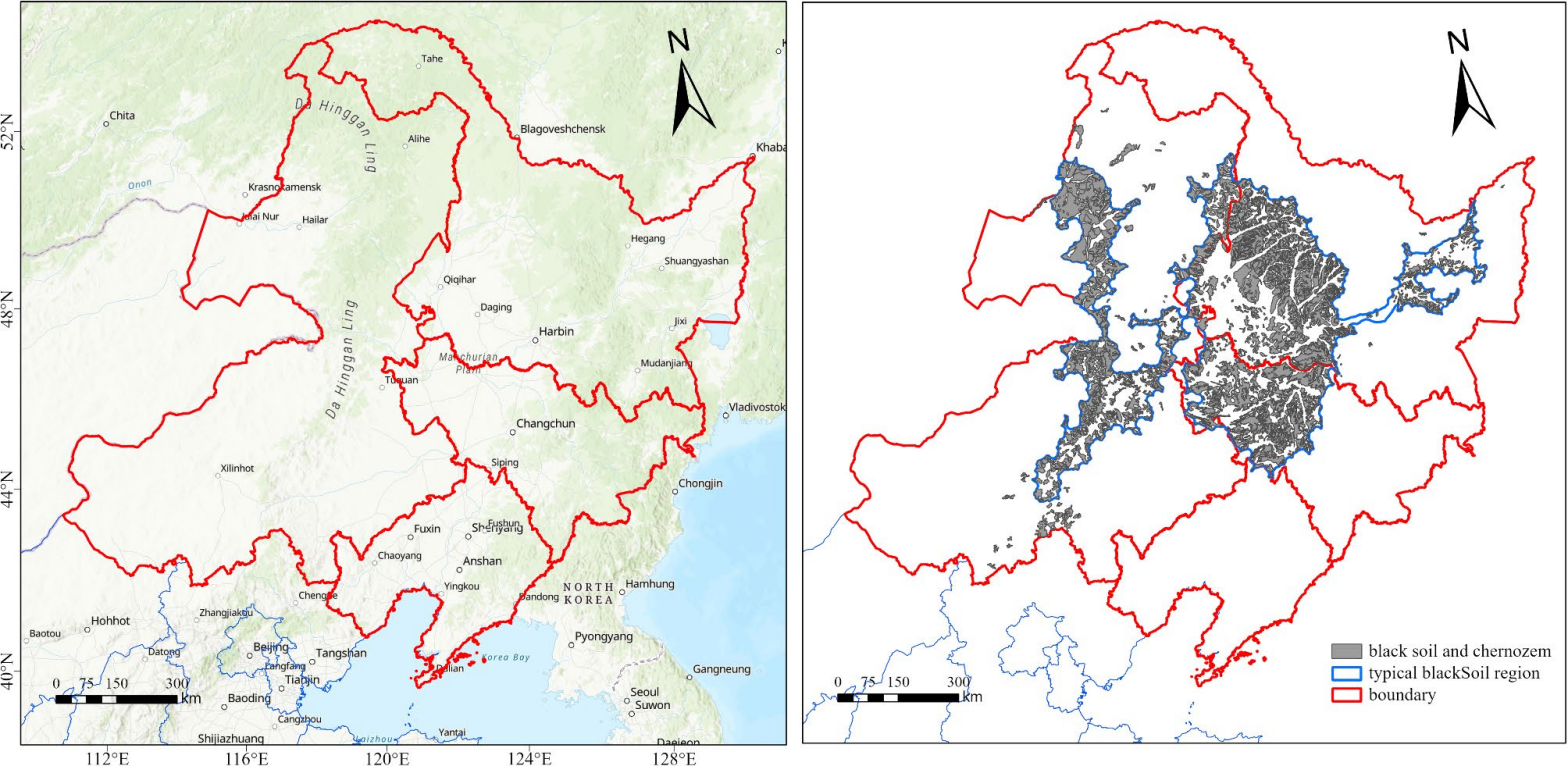
The location of the study area and the scope of black soil in the study area (The background image on the left comes from Esri, OpenStreetMap contributor, TomTom, Garmin, FAO, NOAA, USGS. Map processed by ArcMap 10.8. URL: http://www.esri.com/).

Two different methods are commonly used to define the scope of the black soil region in Northeast China, and the essential difference between them is whether meadow soil is included in black soil. Du et al. (2023) considered that the black soil and chernozem had higher organic matter content, thicker black soil layer and concentrating distribution, and these two types of soil should be classified as typical black soil^50^. According to the above definition of black soil, the scope of black soil region in Northeast China mainly includes most of Heilongjiang Province and Jilin Province, the northern part of Liaoning Province and the northeastern region of Inner Mongolia by attributes extraction and mask processing for data of soil region and cultivated land. Zhao et al. (2020) believed that in addition to black soil and chernozem, meadow soil should also included in black soil, and the digital soil map made by the Institute of Soil Science, Chinese Academy of Sciences was used to determine the scope of three main soil types (black soil, chernozem and meadow soil) in Northeast China^51^, which could serve as the basis for soil classification and black soil region delineation in Northeast China. Considering the fact that the distribution of meadow soil is not concentrating in the study area and cultivated land in the study area is mainly located in black soil and chernozem, the scope of black soil region in this study was finally selected according to the research conducted by Liu et al. (2021) and Wang et al. (2022)^52,53^ (https://geodoi.ac.cn). In the above researches, the definition, spatial distribution and total area of black soil in Northeast China had been explored, and their conclusions could provide reference for determining the scope of black soil region and propose more targeted basis for protecting black soil in Northeast China. In this study, the specific geographic location of the study area and the spatial distribution of black soil and chernozem are shown in Fig. 1:

As shown in Fig. 1, the study area covers the three northeastern provinces and Inner Mongolia Autonomous Region in China. The total area of the study area is 3.330 × 10^5^ km^2^, consisting of black and chernozem with a total area of 1.647 × 10^5^ km^2^. It is worth noting that most of black soil and chernozem in China are located in the study area, accounting for 96.43% and 74.28% of the country’s black soil and chernozem, respectively. The study area is surrounded by mountains on three sides, with plains in the middle, and the Great Xing’an Mountains, Wanda Mountains and Changbai Mountains are the three mountain ranges in the study area. Plains, terraces, hills, and mountains are the main types of landforms, and cultivated land is also mainly distributed here. The study area belongs to a cold temperate monsoon climate with dry and cold climate conditions, and the coldest and hottest month within one year are January and July, with corresponding average temperature locates in − 32 to − 6 ℃ and 20–24 ℃, respectively. The precipitation is abundant and mainly concentrates from July to September with annual average value of 400–700 mm. Black soil and chernozem are the main soil types in the study area, and owing to the unique natural conditions, both the content of soil organic matter and microorganisms is at a high level, resulting in the study area gradually becoming one of the most important commodity grain production bases in China.

### Data and pre-processing

The land use and land cover (LULC) data was used in this study to obtain the scope of black soil region in Northeast China. Specially, the China Land Cover Dataset (CLCD) from 1990 to 2021 published by Yang et al. was selected to determine the scope of black soil region in Northeast China in 1990, 2000, 2010 and 2020^54^. Except for the fine spatial resolution (30 m) of the CLCD product, the accuracy of this product has also been verified. Based on the 5463 visually-interpreted samples, an overall accuracy of 79.31% for the CLCD product can be obtained. Besides, the performance of this product in representing land-use change has been further assessed based on 5131 third-party samples, and the results indicate that overall accuracy of the CLCD product outperforms that of the existing similar products such as MCD12Q1 and GlobeLand30^55,56^. With the help of the CLCD product, land-use types are divided into 9 categories including cultivated land, forest, shrubs, grassland, water, ice and snow, bare land, impermeable water surface (belonging to construction land) and wetland^57^. The administrative zone data are provided by the Resource and Environment Science and Data Center (RESDC), and the boundary data of provincial administration in 2020 and the boundary data of city administration in 2020 were downloaded from the official website of RESDC (https://www.resdc.cn/). The soil types data was also provided by the RESDC and this data was also downloaded from the official website of RESDC in this study. With the help of soil types data, the spatial distribution of soil types in China with a scale of 1:1,000,000 can be obtained. The socio-economic data including population density, total output value of GDP, government residence, road classification data and railway data were used in this study. All selected socio-economic data are derived from official data published by the National Bureau of Statistics, and are obtained by means of the Open Street Map (OSM) in this study. The accuracy and reliability of OSM have been confirmed by the research conducted by Chen et al. (2023)^58^. In this study, the natural environment data including both topographic information and meteorological information were used. For topographic data, the elevation data used in this study was downloaded from the Geospatial Data Cloud (GDC), and then the slope data could be obtained by further calculation. The information for rivers within the study area was also obtained by OSM. For both temperature data and precipitation data were downloaded from the official website of RESDC. All data used in this study are shown in Table 1.

**Table 1:** Data used in this study.

Data	Description	Source	Resolution (m)	Year
Land use and land cover (LULC) data	Typical black soil region in Northeast China	CLCD	30	1990, 2000, 2010 and 2020
Administrative division data	Administrative boundary of province	RESDC	–	2022
Socio-economic data	Gross Domestic Product (GDP)	National Bureau of Statistics (https://www.stats.gov.cn/)	–	1990, 2000, 2010 and 2020
	Population density	Resource and Environmental Science Data Platform (https://www.resdc.cn/)	1000	
	Government residence		–	
	Road	Open Street Map	–	
	Railway		–	
Natural environment data	DEM	Geospatial Data Cloud (https://www.gscloud.cn/)	30	1990, 2000, 2010 and 2020
	Slope	Calculated based on DEM data	30	
	River	Open Street Map	–	
	Temperature	Resource and Environmental Science Data Platform(https://www.resdc.cn/)	1000	
	Precipitation			

Considering that the area of shrubs, ice and snow, and wetlands within the study area is too small (accounting for less than 0.23%), and the main purpose of this study is to analyze the changes in cultivated land, so the land-use types in the study area has been reclassified according to the technical regulations for the Third National Land Survey in China issued by the Ministry of Natural Resources of the People’s Republic of China in 2019. On the basis of the regulations, land-use types in typical black soil region in Northeast China have been reclassified into cultivated land, forest, grassland, water body, bare land and construction land using ArcGIS software. Finally, the land cover datasets of typical black soil region in Northeast China from 1990 to 2020 were obtained. The slope data can be obtained by processing elevation data with the help of slope analysis tool provided by ArcGIS software. Then the accuracy of social and economic factors was verifying, and the cropping process was conducted according to the scope of the study area. It was emphasized that Euclidean Distance Analysis (EDA) was also required for road data, railway data, rivers and government residences within the study area. Finally, all data used in this study were uniformly projected to “Alberts_Conic_Equal_Area” and resampled to 30 × 30 m^2^ spatial resolution by means of ArcGIS software.

### Methods used in this study

### The calculation of graph for geo-information Tupu can be expressed as Eq. (1)

1 $${\text{C}}={\text{A}} \times 10+{\text{B}}$$

where: C is the graph unit attributes of changes during the two adjacent periods, A is the graph unit attribute of the base year of the research period, B represents the graph unit attribute of the end year of the research period. Considering that the time span (1990–2020) in this study is long, graph unit was further calculated according to Eq. (2) to obtain suitable graph unit for this study:

2 $${\text{L}}={{\text{L}}_{1990}} \times 1000+{{\text{L}}_{2000}} \times 100+{{\text{L}}_{2010}} \times 10+{{\text{L}}_{1990}}$$

where: L is the graph unit of changes throughout the entire research period, L_1990_, L_2000_, L_2010_ and L_2020,_ represent the attribute value of land use for each 30 × 30 m^2^ grid.

The Dynamic Degree of Land-use Change (DDLC) index is a reflection of the severity of land-use change, and the DDLC index was used to describe the rate of land-use change within a certain time range quantitatively in this study^59^. The calculation of DDLC index can be expressed as Eq. (3):

3 $${\text{G}}=\frac{{{{\text{G}}_{{{\text{t}}_2}}} - {{\text{G}}_{{{\text{t}}_1}}}}}{{{{\text{G}}_{{{\text{t}}_1}}}}} \times \frac{1}{{{{\text{t}}_2} - {{\text{t}}_1}}} \times 100\%$$

where: G represents the dynamic degree of change in specific land-use type between time t_1_ and t_2_, ${\text{G}}_{{\text{t}}_{\text{1}}}$ and ${\text{G}}_{{\text{t}}_{\text{2}}}$ are the areas of specific land-use type in the base year and the last year of the research period, respectively. It’s worth noting that the fluctuation and positive or negative of G value represent the inflow/outflow situation of cultivated land in the study area, and the larger the absolute value of G, the stronger fluctuation of the land-use change.

The land-use transfer matrix can objectively reflect the transformation relationship between different land-use types, so it is often used to express the mutual transformation between different land-use types quantitatively^60^. In this study, the land-use situation within the study area in different periods can be obtained on basis of geo-information Tupu, and then the land-use transfer matrix in Eq. (4) will be obtained by calculating the area of different land types in different periods:

4 $${\text{S}}_{\text{i}\text{j}}=\left[\begin{array}{ccc}{\text{S}}_{11}& \cdots & {\text{S}}_{1\text{n}}\\ \vdots & \ddots & \vdots \\ {\text{S}}_{\text{n}1}& \cdots & {\text{S}}_{\text{n}\text{n}}\end{array}\right]$$

where: S_ij_ is the area caused by the ${\text{i}}_{\text{th}}$ type of land-use type at the beginning of period to the j_th_ type of land-use type at the end of period, n is the total number of land-use types within the study area, the value for n is taken as 6 in this study.

In this study, both the Changing Rate Index (R) and the Spatial Separation Degree Index (D) were used to analyze the obtained graphs of land-use change during 1990–2020, aiming to reveal the spatial changing characteristics of cultivated land in the study area. The calculation of R index and D index can be expressed as Eqs. (5) and (6):

5 $${\text{R}}_{\text{a}\text{b}}=\frac{{\text{P}}_{\text{a}\text{b}}}{{\sum }_{\text{a}=1}^{\text{n}}{\sum }_{\text{b}=1}^{\text{n}}{\text{P}}_{\text{a}\text{b}}}\times 100\%$$

6 $${\text{D}}_{\text{a}\text{b}}=\left(\text{0.5}\times \sqrt{{\text{A}}_{\text{ab}}/\left({\sum }_{\text{a=1}}^{\text{n}}{\sum} _{\text{b=1}}^{\text{n}}{\text{P}}_{\text{ab}}\right)}\right)/\left(\frac{{\text{P}}_{\text{ab}}}{{\sum }_{\text{a=1}}^{\text{n}}\sum _{\text{b=1}}^{\text{n}}{\text{P}}_{\text{ab}}}\right)$$

where: ${\text{R}}_{\text{ab}}$ is the changing rate, representing the ratio of the area of graph unit after cultivated land change to the total area of all land-use change within the study area. ${\text{D}}_{\text{ab}}$ represents the spatial separation degree of cultivated land conversion, and the larger the value of ${\text{D}}_{\text{ab}}$, the more discrete of the spatial variation of cultivated land. ${\text{P}}_{\text{ab}}$ and ${\text{A}}_{\text{ab}}$ represent the area and number of graph units converted from the a_th_ land type to the b_th_ land-use type, respectively, n is the total number of land-use types within the study area, and the value for n is also taken as 6 in this study.

The Standard Deviation Ellipse (SDE) and Barycenter Model (BM) are jointly used to analyze the changing trend of center of gravity of cultivated land within the study area from 1990 to 2020, aiming to better explore the spatial characteristics of changes in cultivated land utilization. The SDE uses the average and mean square deviation of data to generate an ellipse that can reflect the distribution of data, and can effectively measure the concentrated trend and discrete direction of geographical factors^61^. The calculation of SDE can be expressed as Eqs. (7) and (8):

7 $${\text{E}}_{\text{x}}\text{=}\sqrt{\frac{\sum _{\text{i}\text{=1}}^{\text{n}}{({\text{x}}_{\text{i}}-\stackrel{{-}}{\text{X}})}^{\text{2}}}{\text{n}}}$$

8 $${\text{E}}_{\text{y}}\text{=}\sqrt{\frac{\sum _{\text{i}\text{=1}}^{\text{n}}{({\text{y}}_{\text{i}}-\stackrel{{-}}{\text{Y}})}^{\text{2}}}{\text{n}}}$$

where: ${\text{E}}_{\text{x}}$ and ${\text{E}}_{\text{y}}$ are the standard deviation corresponding to the major and minor axes of the ellipse, respectively. ${\text{X}}_{\text{i}}$ and ${\text{Y}}_{\text{i}}$ are the spatial position coordinate of each feature, ($\stackrel{-}{\text{X}}$, $\stackrel{-}{\text{Y}}$) is the coordinate of the arithmetic mean center. The rotation angle of the ellipse is used to measure the direction of changes in cultivated land. The BM can reflect the spatial evolution process of different land-use types within a specific period effectively, and the calculation of BM can be expressed as Eqs. (9) and (10):

9 $$\text{J}=\frac{{\sum }_{\text{i}=1}^{\text{n}}({\text{j}}_{\text{i}}\times {\text{C}}_{\text{i}})}{{\sum }_{\text{i}}^{\text{n}}{\text{S}}_{\text{i}}}$$

10 $$\text{W}=\frac{{\sum }_{\text{i}=1}^{\text{n}}({\text{w}}_{\text{i}}\times {\text{C}}_{\text{i}})}{{\sum }_{\text{i}}^{\text{n}}{\text{S}}_{\text{i}}}$$

where: J and W are the longitude and latitude coordinates of the center of gravity of spatial distribution for a certain land-use type, respectively, ${\text{j}}_{\text{i}}$ and ${\text{w}}_{\text{i}}$ are the longitude and latitude coordinates of land-use types, ${\text{C}}_{\text{i}}$ represents the area of a certain land-use type.

The PLUS model developed by the National GIS Engineering Technology Research Center of China University of Geosciences (Wuhan) is a cellular automation (CA) model based on grid data, which can be used for simulating land use/cover change at patch scale. The PLUS model integrates the regularly mining method based on land expansion analysis and a CA model based on multi-class random seed mechanism, which has been widely used to explore the driving factors leading to land expansion and predict possible land-use change at patch scale^62^. The implementation of PLUS model mainly includes land expansion analysis strategy (LEAS) and CA model based on multi-class random seeds (CARS) at patch scale. For the LEAS, the expansion of various land-use types between adjacent periods will be extracted firstly, and then the sampling process is conducted for the added portion of land use. Finally, the Random Forest (RF) algorithm is used to mine different driving factors leading to the expansion of various land-use types^63–66^. The principle to determine the impact of different factors on land-use changing using PLUS model can be ex-pressed as Eq. (11):

11 $${\text{p}}_{\text{i}\text{, k}}^{\text{d}}\left(\text{x}\right)\text{=}\frac{\sum _{\text{n=1}}^{\text{M}}\text{I(}{\text{h}}_{\text{n}}\left(\text{x}\right)\text{=d)}}{\text{M}}$$

where: the value of d is 0 or 1 (0 indicates that other land-use types convert to type k, and 1 indicates other transformation types), M means the total number of decision trees contained in a random forest, x is a vector composed of multiple driving factors, I denotes the indicator function of the decision tree set, ${\text{h}}_{\text{n}}\text{(x)}$ represents the prediction result of the ${\text{n}}_{\text{th}}$ decision tree on vector x.

The CARS includes the generation mechanism of a random patch seed based on multiple types of land used/cover. With the help of CARS, the simulated model will be generated automatically based on the probability graph obtained by the LEAS step^67–69^.

### The flowchart of this study is shown in Fig. 2

**Fig. 2:**
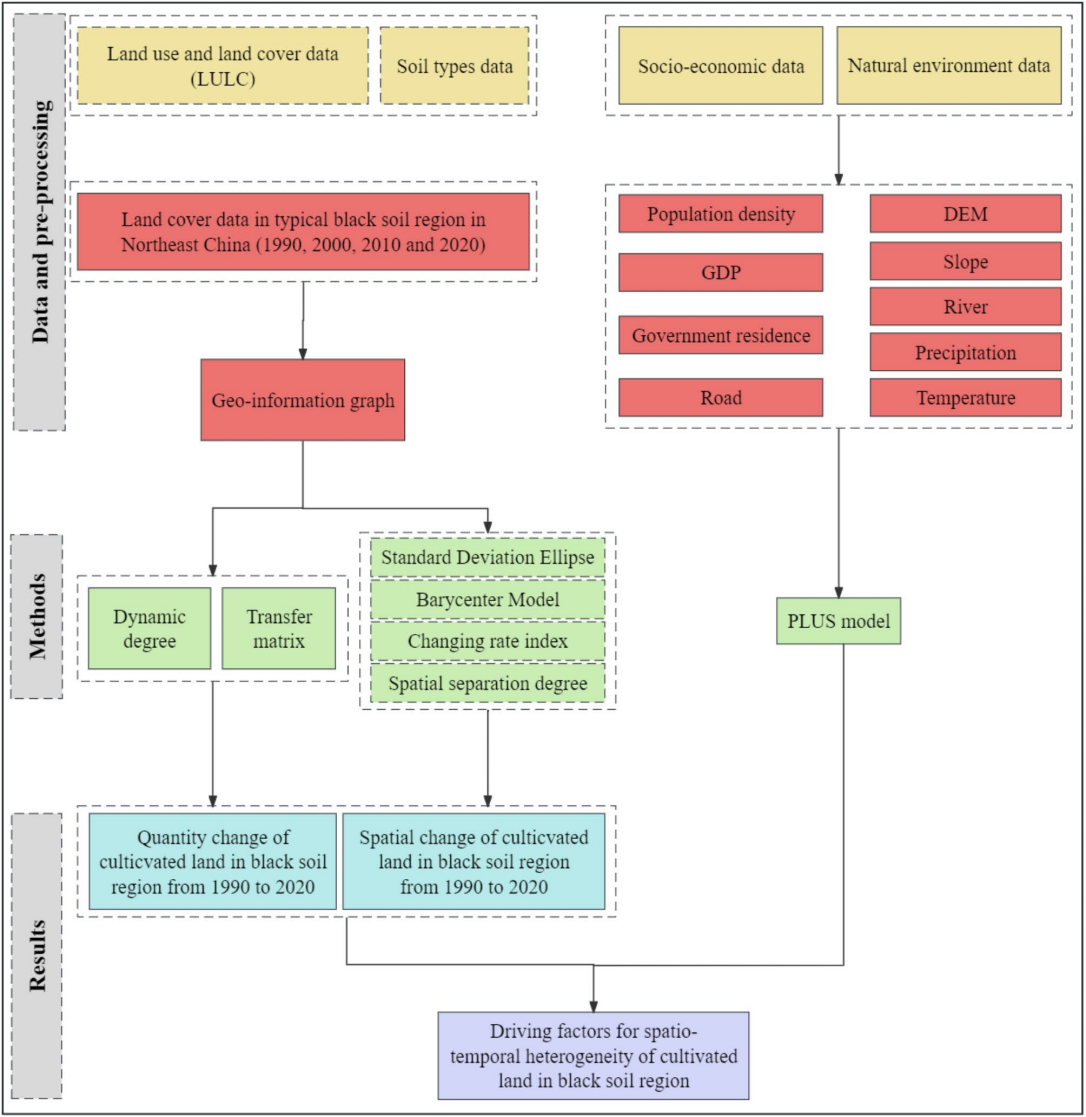
A flowchart of this research.

## Results

### Changes in the quantity of cultivated land utilization

**Fig. 3:**
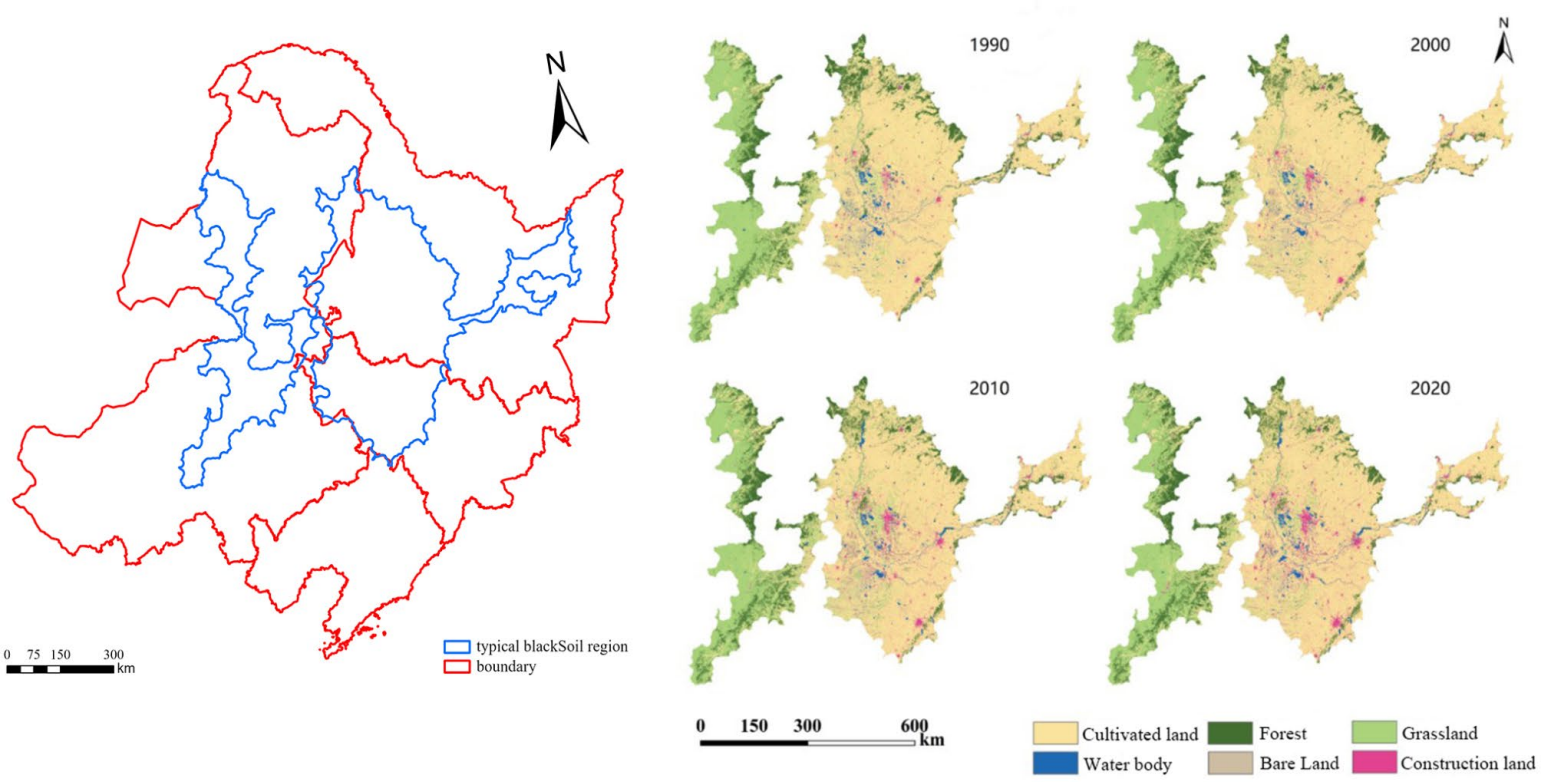
Boundary of the study area and spatial distribution of land-use situation in typical black soil region in Northeast China from in 1990, 2000, 2010 and 2020 (Map processed by ArcMap 10.8. URL: http://www.esri.com/).

Both the embedding and mask process were conducted to the land-use data in 1990, 2000, 2010 and 2020, and then the distribution map of land-use situation in typical black soil region in Northeast China can be obtained and the results are shown in Fig. 3:

As shown in Fig. 3, cultivated land mainly distributes in the eastern part of the study area, that is, concentrating in Heilongjiang Province, Jilin Province and Liaoning Province in China. For the Inner Mongolia Autonomous Region within the study area, cultivated land mainly concentrates in the northern and eastern part and the cultivated land in Xing’an league has a relatively larger proportion. The main land-use types in Inner Mongolia are forest and grassland, and there is also a small amount of grassland distributing in Heilongjiang Province and the grassland is blocky and concentrated at the boundary of the study area. The distribution of water varies significantly over time, mainly concentrating in the central, involving Daqing City, Baicheng City and Songyuan City, and the newly added water mainly located in the northern of Harbin City and at the boarder of Inner Mongolia with Qiqihar City and Heihe City in Heilongjiang Province. To better reflect the changes of different land-use types over the past 30 years, areas of different land-use types in 1990, 2000, 2010 and 2020 were further calculated and the results were shown in Table 2.

**Table 2:** Area of different land-use types within the study area in 1990, 2000, 2010 and 2020 (Unit: km^2^).

Types	1990	2000	2010	2020
Cultivated land	200,737.28	205,969.87	197,542.58	200,865.00
Forest	38,732.68	35,327.35	37,620.32	38,139.89
Grassland	75,827.20	72,065.09	74,778.10	68,008.33
Water	6323.09	4957.00	5372.66	6049.30
Bare land	3390.27	3326.89	2999.11	2183.52
Construction land	7990.41	11,354.74	14,688.17	17,754.90

As shown in Fig. 3; Table 2, construction land is mainly concentrated in the central part of the study area and the area of construction land continued to increase from 7990.41 km^2^ in 1990 to 17,754.90 km^2^ in 2020 (growth rate: 122.20%) over the past 30 years, and the newly added construction land mainly comes from cultivated land. Among the above four periods, cultivated land and grassland account for a relatively large proportion, with a combined area accounting for over 80% of the total area of the study area in each period. It is worth noting that the area of cultivated land is greater than that of grassland, and has shown an “increase-decrease” trend over the past 30 years, leading to a small difference in the total area of cultivated land between the late and early stages of the study. The proportion of bare land in the entire study area is the smallest (with an average proportion of less than 1% in the four periods), with a continuous decreasing trend over the past 30 years and mainly converting to cultivated land and grassland. To further conduct the analysis of the changing characteristics of all land-use types in the study area, the DDLC index was further calculated and the results were shown in Table 3.

**Table 3:** DDLC index of each land-use type within the study area from 1990 to 2020 (%).

Types	1990–2000	2000–2010	2010–2020	1990–2020
Cultivated land	0.261	− 0.409	0.168	0.002
Forest	− 0.879	0.649	0.138	− 0.051
Grassland	− 0.496	0.376	− 0.905	− 0.344
Water	− 2.160	0.839	1.259	− 0.144
Bare land	− 0.187	− 0.985	− 2.719	− 1.186
Construction land	4.210	2.936	2.088	4.073

As shown in Table 3, the DDLC index of different land-use types varies within the same period, and the index of the same land-use type within different periods is also different, indicating different changes of area for different land-use types over the past 30 years. Specially, there has been a significant fluctuation in the area of cultivated land, showing a “increase-decrease” trend over the past 30 years. The grassland has shown a “decrease-increase-decrease” trend. The area of construction land gradually increased and the changing rate gradually slowed down, making it the only land-use type in the study area that continued to increase throughout the entire period. The area of forest and water show a trend of “decrease-increase”, but the total area is still decreasing. The area of bare land continues to decrease but the decline rate gradually accelerates.

**Fig. 4:**
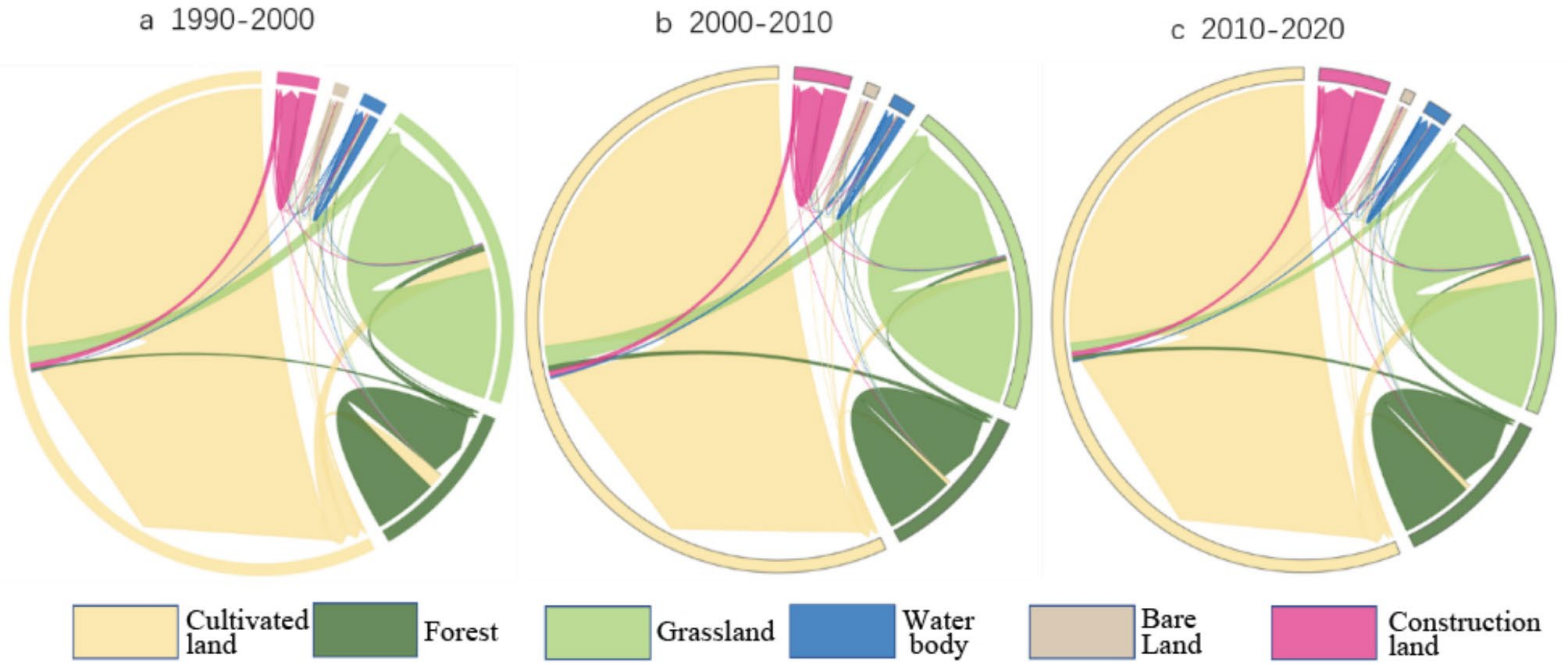
Chord diagrams of land-use transfer matrix from 1990 to 2020 (The area of internal shape represents the area of different land-use types, and the chords with different colors represent the conversions between different land-use types, and the thicker the chord, the larger the conversion area).

**Fig. 5:**
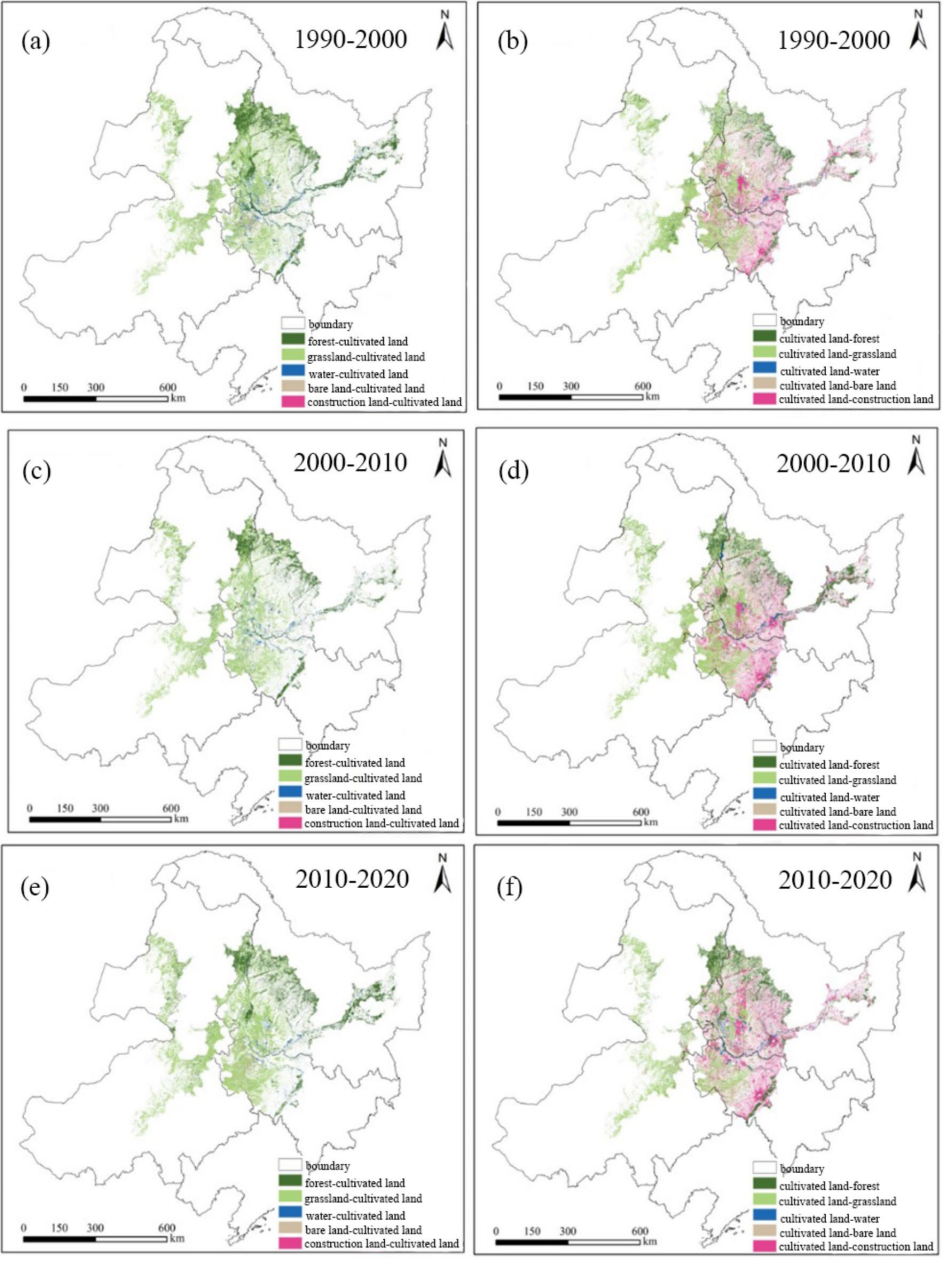
The spatial distribution of RTCL and FTCL from 1990 to 2020 (Note: (**a**) and (**b**) are results during 1990–2000, (**c**) and (**d**) are results during 2000–2010, (**e**) and (**f**) are results during 2010–2020) (Map processed by ArcMap 10.8. URL: http://www.esri.com/).

The rising Tupu of cultivated land (RTCL) infers to the transformation from other land-use types to cultivated land, manifesting a continuous increase of the area of cultivated land within a certain time. While the falling Tupu of cultivated land (FTCL) refers to the transformation from cultivated land to other land-use types, indicating a continuous decrease of the area of cultivated land within a certain time. In this study, the land-use transfer matrix was calculated (Fig. 4), and then both the RTCL and FTCL could be obtained by using the spatial analysis mapping tool provided by ArcGIS software to extract the number of mapping units in land-use transfer matrix. The spatial distribution of RTCL and FTCL from 1990 to 2020 are shown in Fig. 5:

As shown in Figs. 4 and 5a and b, the area of cultivated land increased by 17,596.79 km^2^ from 1990 to 2000, and the conversion from forest and grassland to cultivated land can be responsible for the increase, which account for 41.97% and 49.84% for the increase of cultivated land, respectively. Meanwhile, it can be found that the conversion “forest-cultivated land” is mainly in the northern and eastern regions of the study area, while the conversion “grassland-cultivated land” is mainly located in the central and western regions of the study area. During this period, the area of cultivated land decreased by 12,364.21 km^2^, and most of cultivated land was converted into forest, grassland and construction land, with corresponding values of 1145.85 km^2^, 81,319.99 km^2^ and 2623.24 km^2^, accounting for 9.27%, 65.77% and 21.22% of the total reduction. There are also significant spatial differences in the conversion, namely, the conversion “cultivated land-forest” and “cultivated land-grassland” are mainly in the northeastern and middle, western of the study area. Figures 4 and 5c and d illustrate the change of area for cultivated in 2000–2010 period. The area of cultivated land increased by 7683.75 km^2^, and the increase still mainly come from forest and grassland, and the area caused by the conversion “grassland-cultivated land” is the largest with a value of 5132.37 km^2^, accounting for 66.80% of the total increase of cultivated land. The main regions where grassland converting to cultivated land are still in the central and western region of the study area. There was still a decrease of cultivated during this period, and the total reduction was 16,111.04 km^2^. Compared with the decrease of cultivated land in the previous period, the types of reduction in this period are more diverse, but the conversion “cultivated land-grassland” remains the main reason for the decrease, leading to a 60.63% reduction of cultivated land from 2000 to 2010. As shown in Figs. 4 and 5e and f, the area of cultivated land continued to increase and the grassland and forest contributed the most on the increase. Specially, the area of grassland conversion to cultivated land is 9136.51 km^2^, accounting for 72.20% of the total increase of cultivated land, and the increase caused by the conversion “forest-cultivated land” is 2447.32 km^2^, accounting for 19.34% of the total increase of cultivated land. Compared with the change of cultivated land in the previous period, the diffusion potential increase can be found and the regions with significant growth are mainly located in the central part of the study area. During this period, the decrease of cultivated land was significantly lower than that in the previous two periods, and mainly converted into forest, grassland and construction land. Among all conversion types, the area transferred to grassland is the largest, accounting for 47.21% of the total decrease of cultivated land, and mainly located in the western part of the study area.

**Fig. 6:**
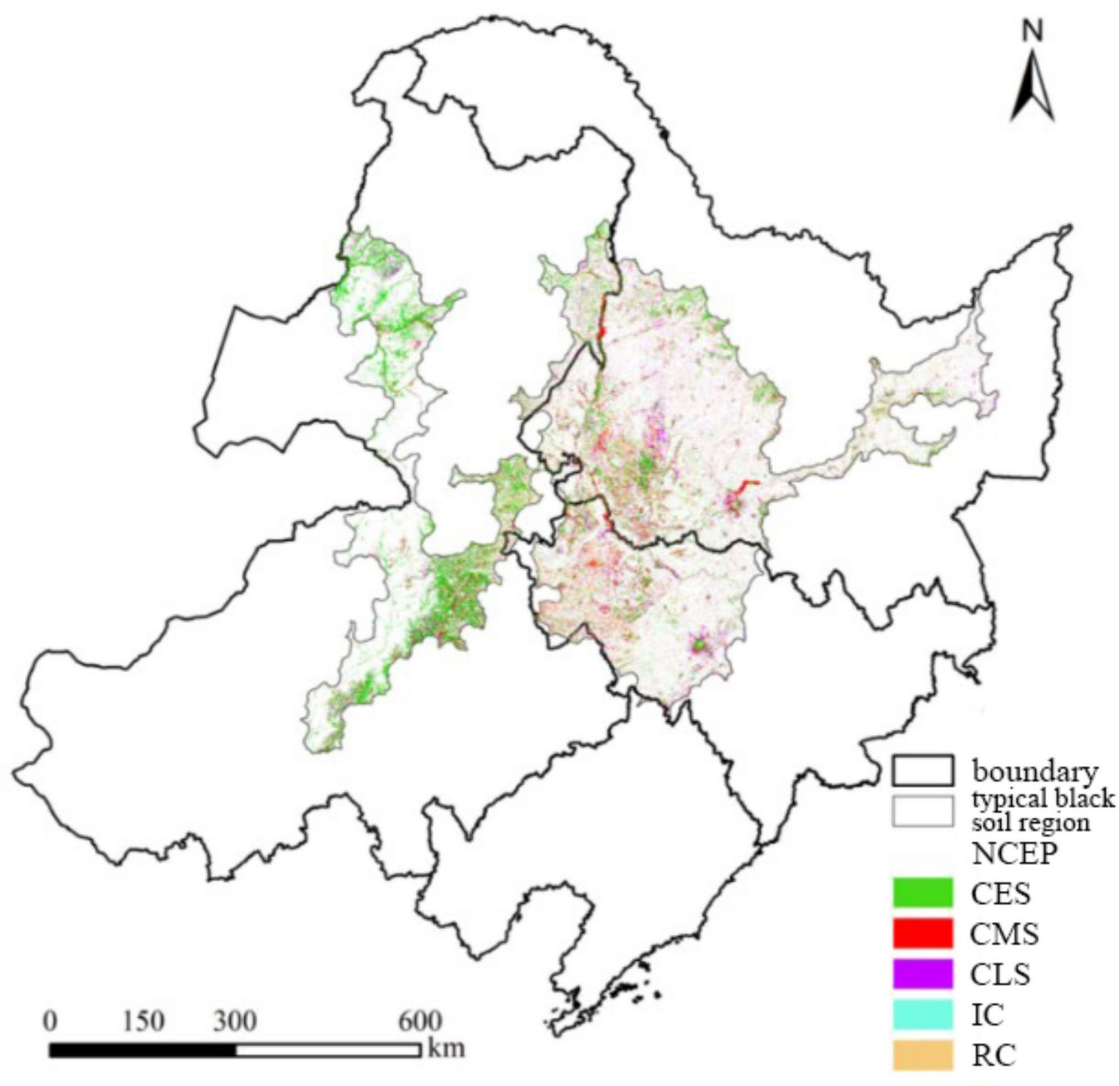
The spatial distribution of 6 different changing patterns during the 1990–2020 period (Map processed by ArcMap 10.8. URL: http://www.esri.com/).

As mentioned above, the entire study period was divided into the first period (1990–2000), the second period (2000–2010) and the third period (2010–2020), and then the changing patterns of cultivated land from 1990 to 2020 could be divided into 6 categories: (1) No change in entire period (NCEP): There was no change in cultivated land in the entire period, (2) Change in early stage (CES): The change of cultivated land only happened in the first period, (3) Change in middle stage (CMS): The change of cultivated land only happened in the second period; (4) Change in late stage (CLS): The change of cultivated land only happened in the third period, (5) Intermittent change (IC): The case that the land-use type in two adjacent periods was cultivated land and (6) Repeated change (RC): The case that the land-use type in the early and late of study period was cultivated land. In order to reveal the changes and spatial distribution characteristics of cultivated land utilization during different periods from 1990 to 2020 intuitively, the area corresponding to the above 6 change patterns and their ratio to the total area of the study area were calculated and the spatial distribution was further mapped. The results are shown in Table 4; Fig. 6.

**Table 4:** Different changing patterns from 1990 to 2020 and the corresponding area and ratio.

Changing pattern	Area (km^2^)	Ratio (%)
NCEP	170,908.47	85.14
CES	7354.92	3.66
CMS	7252.57	3.61
CLS	5753.29	2.87
IC	539.82	0.27
RC	8928.22	4.45

As shown in Table 4; Fig. 6, the proportion of NECP pattern is the highest, accounting for 85.40% of the total area where change of cultivated land happened. Meanwhile, this type of changing is mainly distributed in the eastern part of the study area (mainly in Heilongjiang Province and Jilin Province), and there is also the same change in other regions, extending in a linear pattern from north to the south in the western part of the study area. The area where RC happened follows closely, and the corresponding area is 8928.22 km^2^, accounting for 4.45% of the total study area. As shown in Fig. 6, the RC changing pattern can be found in various regions of the study area including the eastern part of Inner Mongolia and the central region of the study area. The area where CES happened is 7354.92 km^2^ and mainly distributes in the western parts of the study area with a relatively discrete spatial distribution in Hulunbuir City and a concentrated distribution in Chifeng City, Tongliao City, and Xing’an League. The area corresponding to the CMS pattern is not significantly different from the area caused by the CES change, accounting for 3.61% of the total study area with a discrete spatial distribution. The area corresponding to the CLS pattern is 5753.29 km^2^ and the corresponding graph units are mainly located in the eastern part of the study area and in a discrete spatial distribution. The area corresponding to RC pattern is the least with a ratio of 0.27%, and mainly distributed in Tongliao City and Chifeng City, extending in the southwest direction.

### Spatial changes in cultivated land utilization

**Fig. 7:**
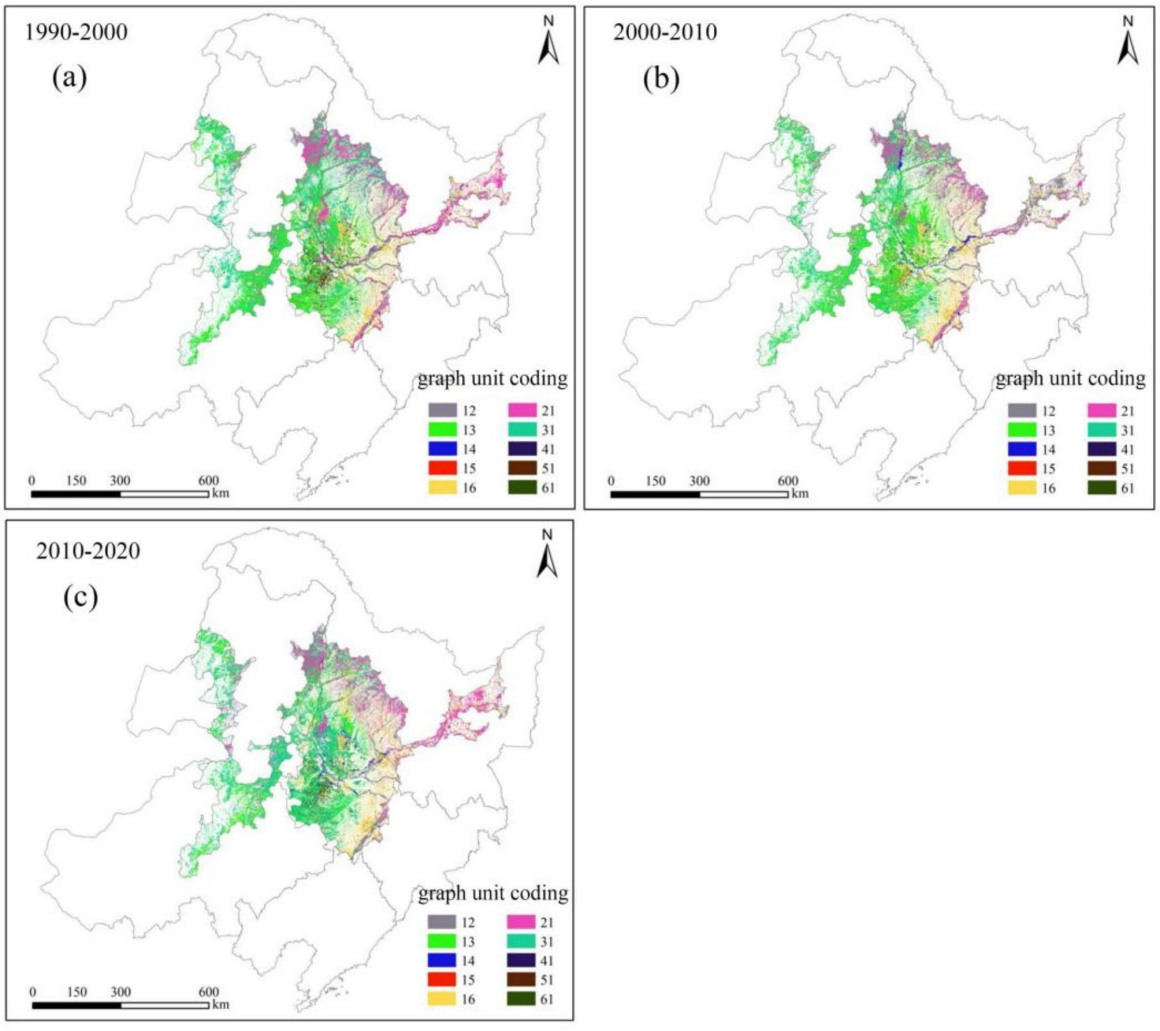
The spatial distribution of different changing types of cultivated land ((**a**–**c**) correspond to 1990–2000, 2000–2010 and 2010–2020 period, respectively. Map processed by ArcMap 10.8. URL: http://www.esri.com/).

To better illustrate the spatiotemporal characteristics of distribution and change of cultivated land utilization, the Changing Rate Index (R) was calculated and the spatial distribution of different types of cultivated land was further mapped. The results are shown in Table 5; Fig. 7.

**Table 5:** Statistics of graph unit representing different changing types of cultivated land. (B, C, F, G, W and CO represent bare land, cultivated land, forest, grassland, water and construction land, respectively).

Period	Type	Count	Area (km^2^)	*R* (%)
1990–2000	G→C	9,745,035	8770.53	4.02
	F→C	8,205,467	7384.92	3.38
	W→C	1,073,502	966.15	0.44
	B→C	521,788	469.61	0.22
	CO→C	6194	5.58	0.003
	C→G	9,035,549	8131.99	3.73
	C→CO	2,914,713	2623.24	1.2
	C→F	1,273,164	1145.85	0.53
	C→W	419,943	377.95	0.17
	C→B	94,637	85.17	0.04
2000–2010	G→C	5,702,631	5132.37	2.40
	F→C	2,129,655	1916.69	0.90
	W→C	529,596	476.64	0.22
	B→C	161,324	145.19	0.07
	CO→C	14,295	12.87	0.006
	C→G	10,852,882	9767.59	4.57
	C→CO	2,853,532	2568.18	1.20
	C→F	2,860,296	2574.27	1.21
	C→W	1,221,847	1099.66	0.52
	C→B	112,596	101.336	0.05
2010–2020	G→C	10,151,681	9136.51	4.35
	F→C	2,719,249	2447.32	1.16
	W→C	368,920	332.03	0.16
	B→C	792,673	713.41	0.34
	CO→C	28,685	25.82	0.01
	C→G	4,895,278	4405.75	2.10
	C→CO	2,495,842	2246.26	1.07
	C→F	2,226,726	2004.053	0.95
	C→W	678,810	610.93	0.29
	C→B	72,973	65.68	0.03

As shown in Fig. 7a; Table 5, the decrease of cultivated land during the 1990–2000 period was mainly caused by the conversion from cultivated land to grassland, construction land and forest. The above conversions are mainly concentrated in the western and southern parts of the study area. Specially, the graph units representing the conversion from cultivated land to construction land are mainly concentrated in the eastern region. But there is significant spatial heterogeneity for the above conversion, the transformation within Daqing City is in a block distribution, and the zonal distribution can be seen in Harbin City, Changchun City and Siping City in the study area. The transformation from cultivated land to forest is mainly located in the eastern region of the study area, and this conversion can also be seen in Shuangyashan City and Qitaihe City in Heilongjiang Province. Meanwhile, the cultivated land to forest conversion in Jilin Province is mainly concentrated in Changchun City in a linear spatial distribution and extend to Siping City. The same conversion in the western part of the study area is mainly in Tongliao City and exhibiting a clustered distribution characteristic. For the increase of cultivated land during this period, the conversion from forest to grassland can be responsible, and mainly can be found in the southwest of Heihe City and the east of Hulunbuir City, followed by the southwest of Jiamusi City and some regions within Shuangyashan City in a linear spatial distribution and extends to Harbin City. In addition, there are graph units clustering in the northwest of Daqing City. The southeastern region of Changchun City also existing the same graph units and extends to the southern region of Siping City. The graph units corresponding to the conversion from grassland to cultivated land are mainly located in some parts within the Inner Mongolia with a clustering spatial characteristic.

During the 2000–2010 period, the conversions from cultivated land to grassland, forest and construction land are the main types leading to the increase of cultivated land. The number of graph units representing the conversion from grassland to cultivated land is the highest, and mainly concentrated in the central and southern regions of the study area. There are also units representing the same changes in the northwest of Hulunbuir City, the west of Suihua City and the west of Xilingol League, and the number of graph units has increased within Xilingol League. The conversion from cultivated land to forest is mainly distributed in the northeast of the study area. The corresponding graph units in Daqing City distributed in blocks and the same units in Jiamusi City and Shuangyashan City can be found in a clustering pattern. Meanwhile, the same changing type of cultivated land in Harbin City and in the east of Suihua City, showing a liner and clustering spatial distribution, respectively. The graph units corresponding to the same change in Hulunbuir City mainly locates in the northwest in a linear pattern, and the number of graph units increases significantly compared with the number in the previous period. The scope where conversion from cultivated land to construction land happened during this period is similar with the scope in previous period and the corresponding number of graph units is not significantly different. The number of graph unit conversion from cultivated land to water has significantly increased compared with the previous period, and mainly distributed in the eastern and northern parts of the study area. The conversion from grassland and forest to cultivated land remains the main factor which can be responsible for the increase of cultivated land, and there is also a small amount of graph units representing the conversion from construction land to cultivated land. The conversion from grassland to cultivated land is generally shifting towards the west, and only concentrates in a few parts within the study area. The graph units corresponding to the conversion from forest to cultivated land has significantly decreased compared with the previous period, and mainly distribute in the northeastern part of the study area, followed by the distribution in southeast of Changchun City and the east of Siping City (Fig. 7b).

**Fig. 8:**
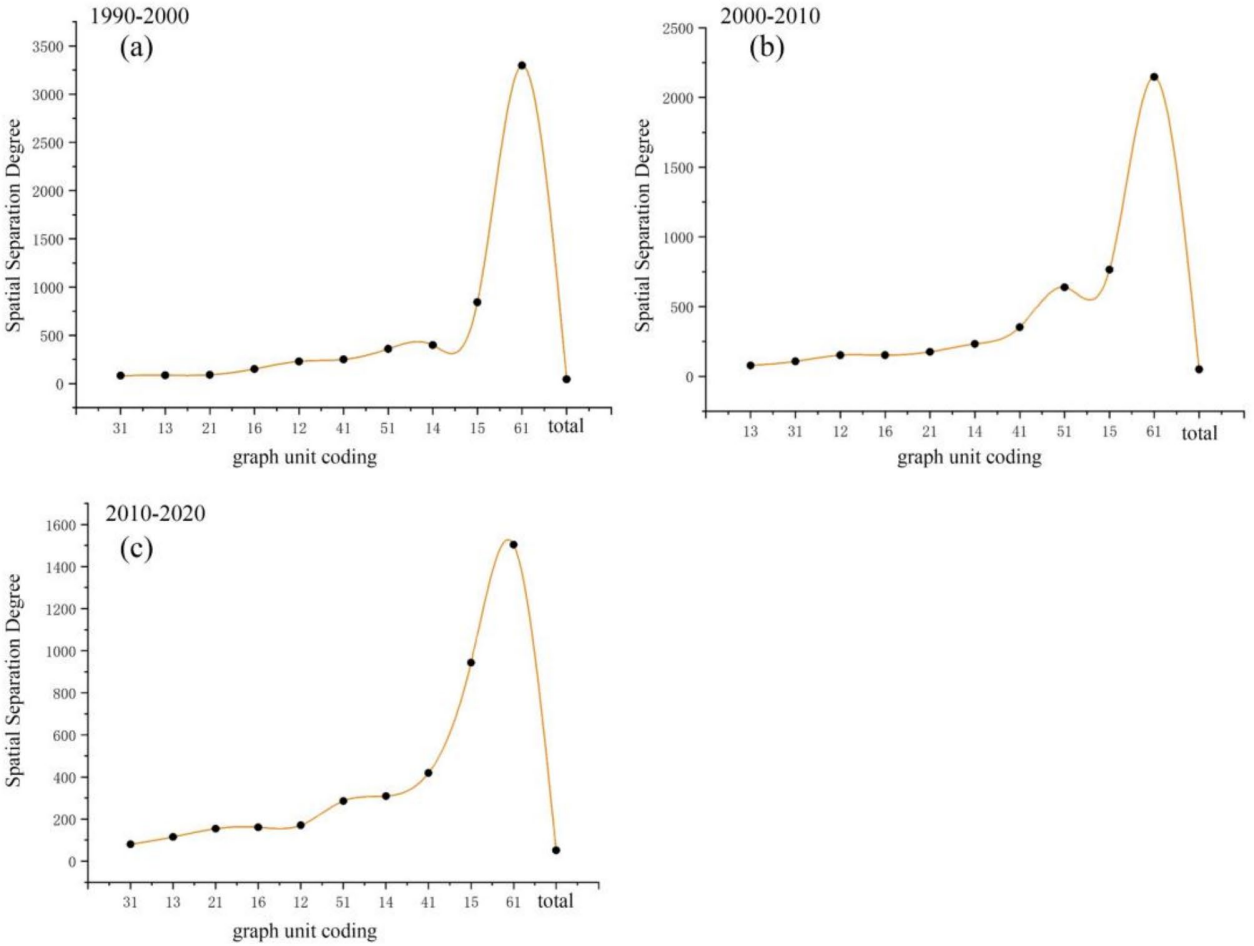
The calculated Spatial Separation Degree Index (D) ((**a**–**c**) correspond 1990–2000, 2000–2010 and 2010–2020 period, respectively).

As shown in Table 5; Fig. 7c, the conversions from cultivated land to grassland land, construction land and forest are the main reasons leading to the decrease of cultivated land during the 2010–2020 period. Among all conversions, the conversion from grassland to cultivated land bring the most reduce of cultivated land and scatters at the junction of Chifeng City, Tongliao City and Xing’an League. There are also graph units corresponding to the above conversion in the northwest of the study area. Compared with the previous period, there has been no significant change in the conversion from cultivated land to construction land in most regions, except for the change in the eastern part of Qiqihar City. The conversion from cultivated land to forest mainly can be found in the northeast and southwest of the study area including the southern and western parts of Heihe City and the southern part of Changchun City. The graph units corresponding to the above conversion also exists in Jiamusi City, Shuangyashan City and Qitaihe City, but the spatial distribution is very discrete and mainly concentrates in the southern part of Shuangyashan City. Except for grassland and forest, the conversion from bare land to cultivated land was another reason leading to the increase of cultivated land. The conversion from grassland to cultivated land mainly can be found in the central part of the study area including. The conversion from forest to cultivated land is mainly distributed in the eastern part of the study area, with very few distributed in the western part. The above conversion is mainly distributed in Harbin City, Qitaihe City and Shuangyashan City in an “F” shape. The spatial distribution of graph units corresponding to the conversion from bare land to cultivated land has hardly changed compared with the previous two periods, and mainly distributes in Baicheng City, Songyuan City and Daqing City. Among all conversions, the number of graph units in Baicheng City is the highest with a “3” shape and extends to the southwest. To reveal the spatial changing characteristics of cultivated land in the study area more intuitively, the Spatial Separation Degree Index (D) is also calculated and the results are shown in Fig. 8.

During the 1990–2000 period, the D indexes corresponding to the mutual conversion between cultivated land and grassland are the lowest, and the difference is small, meaning the significant clustering for the above two conversions (Fig. 8a). Meanwhile, the R index with the value of 3.73% and 4.02% in Table 5 denotes the “as one falls, another rises” relationship between the mutual conversion between cultivated and grassland. The D index corresponding to the conversion from cultivated land to construction land ranks second to the last and mainly concentrated in the central part of the study area. The R indexes corresponding to conversions from cultivated land to water and bare land are 0.17% and 0.04%, respectively, and the corresponding D index is high, meaning the significant discrete feature in space. In addition to the conversion from grassland to cultivated land, the second small D index belongs to the conversion from forest to cultivated land and the corresponding R index is 3.38%. Meanwhile, the R indexes corresponding to the conversion from water to cultivated land and construction land are high and the D value corresponding to the conversion from construction land to cultivated land is 3298.39 with the R index of 0.003%, indicating the higher degree of dispersion and less amount of transfer out. From 2000 to 2010, the R index of the decrease of cultivated land (7.54%) is greater than that of the increase of cultivated land (3.596%). The D index corresponding to the conversion from cultivated land to grassland is 77.95, indicating the enhanced clustering of the conversion compared with the previous period. The R and D indexes for the conversion from cultivated land to construction land are 151.54 and 1.20%, respectively, which are similar with the values in the previous period. The D index for the conversion from cultivated land to forest is 151.54 which reduced significantly compared with the previous period but the change of D index denotes the reduced dispersion. The R indexes for the conversion from bare land and water to cultivated land are 0.05% and 0.52%, respectively, and the corresponding D indexes are 765.28 and 232.31, respectively, indicating the enhanced clustering of conversion. The R index for the conversion from grassland to cultivated land is highest and the corresponding D index during this period is small, leading to the more discrete conversion. The R indexes corresponding to the conversion from forest and water to cultivated land have both decreased, and the degree of spatial dispersion has intensified. The R index from bare land to cultivated land has decreased during this period with a D index of 639.34, meaning the distinct discrete characteristic. The R index for conversion from construction land to cultivated land has increased compared with the previous period, but it still accounts for the smallest proportion of all conversions with a spatial separation degree of 2147.78.

As shown in Table 5; Fig. 8c, the R index decreased from 11.14 to 10.46% with a relatively stable change. The R and D indexes corresponding to the conversion from grassland to cultivated land are 2.10% and 115.12, respectively, indicating the more aggravated dispersion compared with the two previous periods. The D index for the conversion from cultivated land to forest is 170.69, meaning the increasing degree of separation compared. The R index for the conversion from cultivated land to construction land has decreased and the corresponding R index has increased significantly during this period. The D indexes corresponding to conversions from cultivated land to water and bare land are 309.15 and 942.89, respectively, denoting the distinct spatial dispersion of the conversions and the inactive characteristic. The R index of grassland and forest to cultivated land is relatively high, but the D index is the lowest, with the value of 79.94 and 154.46, respectively, indicating the rich conversion types resulting in the increase of cultivated land during this period and the high spatial clustering characteristic. The sum of the R index for the conversion from construction land and water to cultivated land is only half of the R value for the conversion from bare land to cultivated land, but the D index for the conversion from construction land to cultivated land is high, indicating the highest degree of dispersion.

**Fig. 9:**
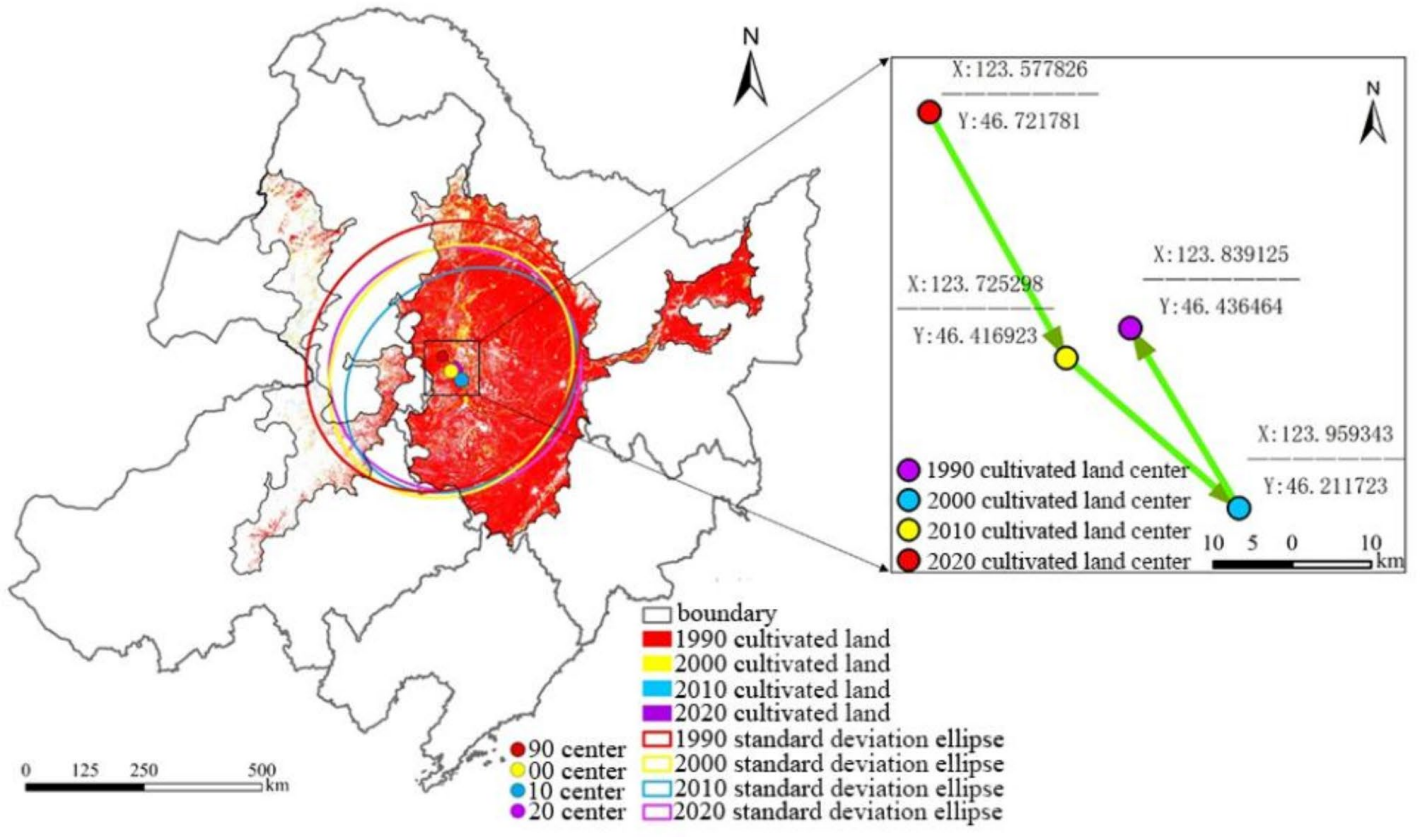
The spatial distribution of obtained SDE and BM within the study area from 1990 to 2020.

The ArcGIS software was utilized to calculate the parameters of SED (nearly covering 68% of the entire cultivated land within the study area) and BM to obtain the spatial evolution process of different land-use types within the study. The results are shown in Table 6; Fig. 9.

**Table 6:** The parameters for the SED in 1990, 2000, 2010 and 2020.

Year	CenterX (m)	CenterY (m)	XStdDist (m)	YStdDist (m)	Rotation (°)
1990	1,410,480.60	5,186,884.43	304,824.20	269,790.20	48.07
2000	1,427,908.99	5,155,645.12	244,417.20	280,061.80	37.788
2010	1,449,880.75	5,136,579.29	265,288.50	216,928.30	49.950
2020	1,436,074.20	5,159,425.06	268,027.30	256,065.50	71.263

As shown in Fig. 9, there is a significant change in the spatial distribution of cultivated land utilization in the past 30 years (1990–2020). Specially, the oblateness of SED increased from 1990 to 2010, indicating that there was a clear directional trend of the change of cultivated land. However, the oblateness of SED sharply decreased in 2020, denoting that the directional trend was not obvious. Both the major and minor axes of SED showed a decreasing trend during this period. The major axis decreased from 304.82 km in 1990 to 268.03 km in 2020 with a significant decreasing trend, indicating that the degree of dispersion in the spatial distribution of cultivated land utilization has gradually increased. The minor axis of decreased from 269.79 km in 1990 to 256.07 km in 2020, demonstrating the more centripetal trend of cultivated land utilization. The transfer trajectory of BM during the 1990–2020 period in Fig. 9 indicates that the core of cultivated land migrated to the southeast from 1990 to 2010, and the migration distance during 1990–2000 period and 2000–2010 period were 35.77 km and 20.09 km, respectively. Then the core of cultivated land showed a convoluted trend and migrated to the northeast with a distance of 26.69 km.

### Driving factors for changes of cultivated land utilization

**Fig. 10:**
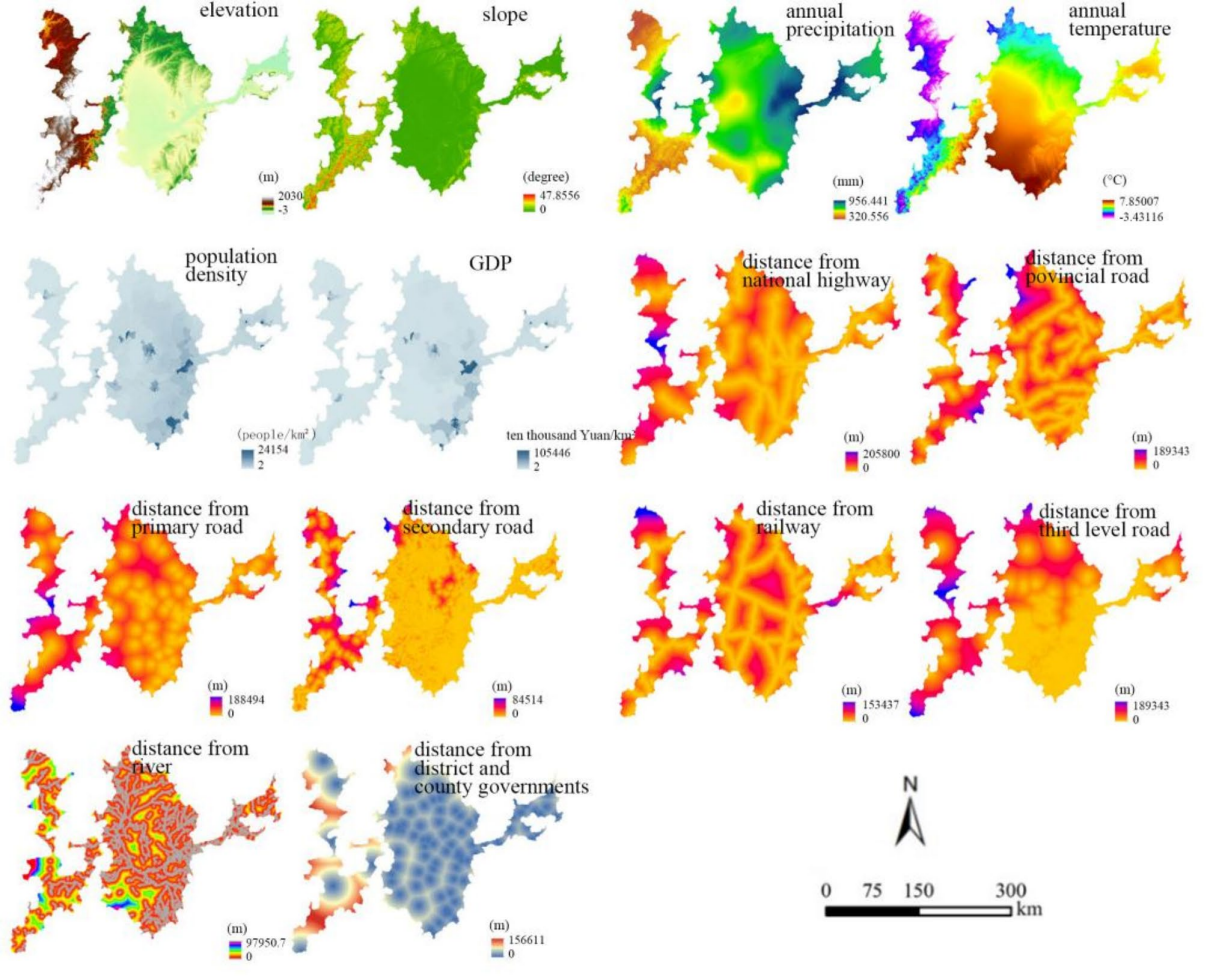
Raster graphs for different driving factors in typical black soil region in Northeast China.

**Fig. 11:**
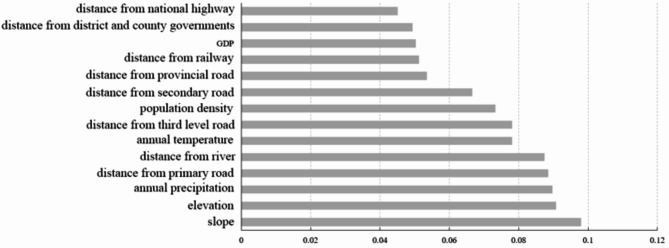
Contributions of different driving factors on the expansion of cultivated land from 1990 to 2020.

Compared with other models, the PLUS model increases its understanding of the underlying nonlinear relationships for land-use change, and is better at investing the potential conversion rules for the change^70^. The excellent performance of PLUS model in simulating land-use change and analyzing the driving factors for land expansion has been validated in many previous studies^71,72^. In this study, the PLUS model was utilized and natural environment factors including elevation, slope, precipitation, temperature, river and socio-economic factors including population density, GDP, distance from road, government residences were selected to analyze the driving factors for the change of cultivated land utilization in typical black soil region in Northeast China. To reduce data redundancy and improve efficiency, a resampling process was firstly conducted to the data for land-use expansion and prediction, and then the raster data at 90 × 90 m^2^ was obtained. Subsequently, the land-use data in 1990 and 2020 and driving factor data were projected to the “Albers_Conic_Equal_Area” coordinate system by means of the ArcGIS software. The resampling operation enables better matching and comparison between different data, while reducing the impact of random errors and outliers in data. With the help of resampling, data size can be adjusted to the suitable size for model operation, aiming to reduce the dimensionality and computational complexity and improve the running efficiency of the model without losing important information. Meanwhile, the unification of spatial reference frames for different data can ensure the accuracy of change for cultivated land use, and guarantee the precision of the model’s results. Next, the Euclidean Distance Analysis was performed on the river data, road data and government residences data, and the raster graph of distance factor can be obtained. Owing to its own advantage, the Euclidean Distance Analysis method can accurately quantify the spatial relationship between driving factors and cultivated land, which can be used to provide intuitive explanations for the impacting degree of driving factors. By the results of Euclidean Distance Analysis, the impacting range and intensity of different driving factors can be obtained directly. Finally, the environment variable was set and the mask extraction was conducted to the raster data according to the row and column numbers of land-use data in the study area, aiming to make the required data and prediction data have the same row and column numbers and obtain the driving factors in raster graph. The study area can extracted from the entire raster images after implementing the mask extraction, and all irrelevant regions can be eliminated, leading to the improvement of computational efficiency. The obtained raster graphs for different driving factors are shown in Fig. 10:

The LULC data in raster form in 1990 and 2020 were selected and the overlay process was conducted on the selected data to determine the number of grids corresponding to the expansion of different land-use types. According to the technical regulations for the third national land survey in China and considering the small areas of shrubs, ice and snow, and wetlands, and the fact that the changes of cultivated land are focused in this study, so the reclassification tool embedded in ArcGIS software was used to divide the land in the study area into cultivated land, forest (including shrub and wetland), grassland, water body (including ice and snow), bare land and construction land. On the basis of establishing the classification system, the reclassification operation of land use for the typical black soil region in Northeast China was conducted. Then the extracted expansion data and driving factor data were added to the LEAS module embedded in PLUS model, and the sampling rate and decision tree parameters were set to default values as 0.01 and 20, respectively. Due to the fact that the number of features for training RF is less than the number of driving factors, so it is set to 14 and the number of parallel threads was set to 8. The residual root mean squared error (rRMSE) for cultivated land, forest, grassland, water, bare land and construction land are 0.163, 0.098, 0.128, 0.067, 0.048 and 0.106, respectively, demonstrating the excellent operating result of the PLUS model. Meanwhile, the simulated results in 2020 by the PLUS model was compared with the actual land-use situation in 2020, and then both the Kappa coefficient and overall accuracy (OA) were calculated. Compared with previous studies using the PLUS model whose Kappa coefficient are 0.765 and 0.817, respectively, the obtained Kappa coefficient of 0.828 and OA of 0.898 demonstrated the high reliability of the results for PLUS model used in this study^73,74^. Then the probability graph of the development of different land-use types and the contributions of different factors on the change of cultivated land utilization during this period can be obtained. To analyze the impact of driving factors on cultivated land change, the raster graphs of cultivated land increase and driving factors from 1990 to 2020 were overlaid, and the contribution of different driving factors on the expansion of cultivated land during this period was shown in Fig. 11:

As shown in Fig. 11, the main contributing factors to the increase of cultivated land during the study period are slope, elevation, annual precipitation and distance to the primary road. By overlaying the increasing area of cultivated land with the raster data of slope, it can be found that the increasing scenario of cultivated land are usually distributed in regions with relatively gentle slopes. However, due to the high elevation throughout the Inner Mongolia, the increase of cultivated land within Inner Mongolia are mainly concentrated in regions including the eastern part of Xing’an League and Hulunbuir city with relatively low elevation, and there is usually a large amount of precipitation in the above regions which is suitable for crop growth. During the 1990–2020 period, the increase of cultivated land is 9332.67 km^2^, among which the proportion from cultivated land to forest, grassland, construction land is 21.47%, 47.21% and 24.07%, accounting for 63.58%, 93.33% and 64.90% of the increase of forest, grassland and construction land, respectively, demonstrating the expansion of these three land-use types are the main factors responsible for the decrease of cultivated land within the study area during the 1990–2020 period.

## Discussion

### The comparison of Temporal change of cultivated land utilization

In this study, the area change of cultivated land utilization within the study area were obtained based on graph representing different types of land-use change during the 1990–2020 period. The results indicate that the cultivated land has the greatest fluctuation from 1990 to 2020, which is a relatively active land-use types during this period, and this conclusion is consistent with the findings obtained by Li et al.^65^. Both the changing graphs and transfer matrix of cultivated land utilization indicate that the conversion from cultivated land to forest, grassland and construction are the main means leading to the decrease of cultivated land during the 1990–2020 period, while the conversion from forest, grassland to cultivated land can be responsible for the increase of cultivated land during this period. The reason for the above situation may be attributed to the implementation of protection policies at different periods. Specially, in order to meet the increasing demand for food, government encourages people to plant a large amount of food crops in the study area, resulting in a considerable portion of forest and grassland have been reclaimed as arable land. However, with the sharp decrease in forest and grassland, the ecological environment has deteriorated and intensified soil erosion. In order to address the above issues, the Chinese government proposed a series of forestry protection measures such as the “Natural Forest Protection Project” in the late 1990s, aiming to increase the area of forest in the study area. As one of the important state-owned forest regions in China, a series of effective afforestation activities have been carried out in the study area and a large amount of abandoned cultivated land has been converted into forest. In recent years, as food security issues have gradually gained attention, the implementation of a series of protective policies has ensured the increase of the area of cultivated land and the stability of food production, and the above situation is more evident in the Northeast Plain of China. As an important grain producing region in China, maintaining the area of cultivated land in the Northeast China is of great strategic significance. The above reasons have jointly led to an “increase-decrease-increase” trend in the cultivated land in the study area. However, the total area of cultivated land did not change significantly between 1990 and 2020, with only a net increase of 127.72 km^2^, demonstrating the effectiveness of balancing the relationship between development and protection of cultivated land over the past 30 years.

### The comparison of Spatial heterogeneity of cultivated land utilization

The analysis of the spatial distribution and change of cultivated land utilization indicates that the spatial separation of cultivated land continued to expand from 1990 to 2020. The main reason for the above situation is that there are significant differences in the spatial distribution of land-use types within the study area, and the utilization and protection policies of cultivated land vary among different provinces in the study area. By comparing the changing rate of cultivated land in 3 different periods, it can be found that the graph units with the highest spatial distribution in the study area are the ones that representing the conversion between cultivated land, forest and grassland, and the construction land has almost no “return” on cultivated land, but only “demand”. In addition to cultivated land, construction land also encroaches on a certain amount of forest and grassland, not only having a negative impact on food security, but also causing damage to the ecological environment. The conclusion of this study further confirms that the government should strengthen the control of cultivated land and adhere to the bottom line to protect basic cultivated land strictly. Meanwhile, the center of cultivated land is generally moving towards the southeastern direction in the study area from 1990 to 2020, demonstrating a trend of gradual concentration of cultivated land in this direction. As a provincial administrative region with abundant forest resources, the “Grain for Green” policy has been taken in Heilongjiang Province, leading to the conversion from cultivated land to forest, which may be responsible for the move of center of cultivated land. But it’s worth noting that the migration distance gradually slowed down, indicating that the magnitude of changes in cultivated land tended to moderate.

## Conclusions

In this study, the spatiotemporal changes of cultivated land in black soil region in Northeast China from 1990 to 2020 were analyzed comprehensively and the driving factors for the spatiotemporal heterogeneity over the past 30 years was explored subsequently. The area of cultivated land in typical black soil region in Northeast China only increased by 127.72 km^2^ (accounting for 0.038% of the study area) with a changing trend of “increase-decrease-increase”. During this period, cultivated land was mainly converted to forest, grassland and construction land, and the conversion from forest, grassland to cultivated land can be responsible for the increase of cultivated land, demonstrating the fact that changes of land-use policies can directly lead to variations in cultivated land area, but the bottom line of cultivated land area still need to be maintained to ensure the food production security. Although the area of cultivated land in most of the black soil region in Northeast China has shown a long-term unchanging trend, the degree of spatial separation of cultivated land has still increased owing to the differences in land-use policies between different provinces within the study area. Meanwhile, the barycenter of cultivated land has gradually shifted towards to southeast over the past 30 years, indicating a continuous increase in the density of cultivated land in this direction, but the changing trend is gradually slowing down. The change of cultivated land in Northeast China over the past 30 years are the results of the combined effect of natural factors and socio-economic factors. As is well known, the yield of crops grown in cultivated land will directly affect by natural factors including slope, elevation and precipitation. Especially in the current context of global warming, changes and spatial difference in natural factors will inevitably lead to the spatiotemporal heterogeneity in cultivated land use. Meanwhile, the impact of socio-economic factors on cultivated land cannot be ignored, and the relationships between distance from roads, distance from rivers, population distribution and cultivated land use has been demonstrated in this study. Specially, the distance from roads and rivers will directly affect the convenience of land cultivation and irrigation, while the population distribution is determines whether there are sufficient human resources to complete cultivation and management. Conversely, the construction of road and the expansion of residential area can also lead to the reduction in cultivated land area. In summary, the conclusion of this study will provide referencing significance for the formulation of land-use plans in Northeast China and ensuring the food security in China.

## Data Availability

Data is provided within the manuscript or supplementary information files.
